# The strategic distraction motive: addiction as attentional regulation under perceived insolubility

**DOI:** 10.3389/fpsyg.2026.1829035

**Published:** 2026-05-29

**Authors:** Funso Akinola Alaye, Omar Gelo, Human-Friedrich Unterrainer

**Affiliations:** 1 Faculty of Psychotherapy Science, Sigmund Freud University Vienna, Vienna, Austria; 2 Department of Human and Social Sciences, University of Salento, Via di Valesio SNC, Lecce, Italy; 3 ARH: Addiction Research Hub, Grüner Kreis Ltd., Vienna, Austria; 4 Department of Religious Studies, University of Vienna, Vienna, Austria; 5 Department of Psychiatry and Psychotherapeutic Medicine, Medical University of Graz, Graz, Austria

**Keywords:** addiction, attentional control, attentional processes, cue-reactivity, ecological momentary assessment, emotion regulation, experiential avoidance, insolubility

## Abstract

Contemporary models of addiction provide powerful accounts of reinforcement learning, cue-reactivity, affect regulation, and defensive processes. Yet they remain comparatively underspecified regarding a recurrent clinical phenomenon: the micro-temporal conditions under which attention reorganizes from engagement with activated experience to structured reorganization into addiction-related behavior or discourse, particularly during moments of relational proximity, identity threat, or symbolic strain not reducible to distress magnitude or cue exposure alone. This article proposes the Strategic Distraction Motive (SDM) as a process-level mechanism specifying a within-person transition condition for such shifts. SDM posits that when activated experience is appraised as insoluble, that is, not effectively engageable, articulable, or resolvable within current symbolic, regulatory, or relational capacities, attentional organization reallocates toward a bounded and socially legible addiction-related domain. This reallocation preserves short-term experiential coherence and interpersonal organization while deferring engagement with the precipitating threat. The model advances three discriminant predictions: (1) within individuals, momentary increases in perceived insolubility will prospectively predict addiction-focused attentional pivots above and beyond craving intensity, negative affect, cue exposure, and experiential avoidance; (2) such pivots will exhibit content specificity relative to generic distraction; and (3) addiction-framed pivots will systematically reorganize interpersonal responding. SDM formalizes a temporally ordered sequence linking appraisal, attentional substitution, and relapse clustering, thereby reframing addiction not solely as dysregulation or compulsion, but as context-sensitive attentional governance under constraint. By specifying timing, mechanism, and discriminant tests, SDM aims to increase theoretical precision while remaining empirically tractable and scope-bounded.

## 1 Introduction

Neurobiological models conceptualize addiction as dysregulation in reward, motivation, and executive control systems involving sensitization, habit learning, and impaired control processes (Koob and Volkow, 2016). Cognitive-behavioral and relapse-prevention frameworks highlight affect modulation, coping deficits, and high-risk contexts (Marlatt and Donovan, 2005), whereas psychodynamic accounts describe defensive processes such as denial and dissociation under threat (Vaillant, 1995), whereas self-medication formulations emphasize affect regulation and compensatory substance use (Khantzian, 1997). Together, these traditions provide robust accounts of vulnerability, maintenance, and relapse risk.

At the same time, clinical observation and adjacent literatures suggest that relapse risk and substance-focused engagement fluctuate within individuals across relational and meaning-level contexts, including moments not reducible to craving intensity or negative affect alone. This inference draws on experience-sampling methods (Shiffman et al., 2008), dynamic relapse models (Witkiewitz and Marlatt, 2004; Witkiewitz et al., 2005), psychotherapy process accounts of threat-related interactional shifts (Safran and Muran, 2000), attachment research on proximity-linked regulatory changes (Mikulincer and Shaver, 2016), and recent intensive longitudinal work emphasizing within-person variability in substance use processes (Cleveland et al., 2023; Knapp et al., 2025). In practice, these fluctuations are often experienced as temporally precise pivots toward substance use or addiction-focused discourse that may concentrate around moments of relational proximity, identity threat, evaluative exposure, or symbolic breakdown rather than distress magnitude alone. These observations do not contradict existing models; rather, they raise a more specific timing question: under what within-person conditions does attention reorganize toward addiction-related domains in particular moments? Such an emphasis on relational and process-level phenomena is coherent with broader mappings of psychotherapy research according to which the clinical relationship and clinical efficacy represent central topics in the field (Gennaro et al., 2019; Lingiardi et al., 2016).

The Strategic Distraction Motive (SDM) is proposed as a process-level specification of this timing question. SDM conceptualizes addiction-related behavior or discourse as a form of attentional substitution recruited when activated experience is appraised as insoluble within available symbolic, regulatory, or relational capacity. Under such conditions, attention is hypothesized to shift away from destabilizing material toward a bounded, socially legible addiction-related domain. Using Goffman (1959) as a theoretical scaffold, the addiction frame is treated as a culturally recognizable interactional script that can organize interpersonal responding and preserve short-term coherence without resolving the precipitating threat.

Importantly, SDM does not dispute reinforcement-based, cue-reactivity, affect-regulation, or attachment-based accounts. Its contribution is narrower and process-specific: to formalize a candidate within-person transition condition, perceived insolubility, under which attentional organization shifts into addiction-related domains. By isolating attention as a regulatory medium, SDM seeks to increase precision regarding when and how addiction-related engagement is deployed in context, rather than to replace existing vulnerability or maintenance models.

Thus, although stress and contextual influences are well documented, the field remains comparatively less explicit about the micro-temporal conditions under which attentional regulation shifts from engagement with activated experience to addiction-related substitution (Curran and Bauer, 2011; Kazdin, 2007).

### 1.1 Why existing models under-specify attentional timing

Contemporary theories of addiction provide powerful explanations of vulnerability, maintenance, and relapse risk. Neurobiological models detail incentive sensitization, habit learning, and dysregulated control circuitry (Koob and Volkow, 2016), while cognitive-behavioral and relapse-prevention frameworks emphasize affect modulation, coping processes, and high-risk contexts (Marlatt and Donovan, 2005). Attachment-based and psychodynamic accounts describe relational activation and defensive processes under threat (Mikulincer and Shaver, 2016; Vaillant, 1995). Together, these traditions offer robust and complementary accounts of how addictive behavior becomes established and sustained.

However, this literature is comparatively less explicit about when, within individuals, attentional organization shifts from engagement with activated experience to substitution into addiction-related domains. Neurobehavioral and relapse-prevention models account for cue-reactivity, craving dynamics, stress reactivity, and contextual risk factors, and some extensions incorporate interpersonal determinants of relapse; however, these accounts typically treat such influences as antecedents or moderators of craving and affect rather than specifying a process by which appraisal of experience reorganizes attention toward addiction-focused cognitive or behavioral domains (Epstein et al., 2009; Hendershot et al., 2011).

Recent intensive longitudinal and computational work further suggests that short-lag transitions in substance use are heterogeneous within persons and may reflect multiple interacting pathways rather than a single linear sequence (Cleveland et al., 2023; Gauld et al., 2025). Thus, although stress and contextual influences are well documented, the field remains comparatively less explicit about the micro-temporal conditions under which attentional regulation shifts from engagement with activated experience to addiction-related substitution.

Affect-centered formulations compellingly demonstrate that substances modulate distress, yet they frequently treat distress magnitude as the primary driver of use. Research on emotion regulation complicates this linear assumption, showing that attentional deployment is selected not solely as a function of affect intensity but also of perceived regulatory feasibility and cognitive cost (Gross, 2015). These findings imply that regulatory shifts are appraisal-sensitive; however, the addiction literature has not consistently operationalized or tested appraisal-contingent, micro-temporal attentional transitions as a distinct within-person process, leaving the timing question comparatively underspecified. Similarly, experiential avoidance frameworks conceptualize substance use as escape from unwanted internal experience (Hayes et al., 1996). However, within this literature, avoidance is often treated at a broad functional level, even though particular avoidance patterns can be linked to specific experiential domains. These accounts do not generally predict why attention would preferentially reorganize toward addiction-related domains rather than toward neutral distractions, nor do they specify observable micro-temporal markers of such transitions. Trait-based vulnerability models identify between-person risk factors such as insecure attachment or low perceived control (Bowlby, 1988; Mikulincer and Shaver, 2016), but they do not articulate the within-person sequence by which situational activation translates into addiction-focused attention in particular moments.

Taken together, these literatures converge on a shared limitation: they describe why addiction persists but not when attentional regulation shifts from engagement to substitution. Consistent with mechanism-focused standards emphasizing temporal ordering, mediating processes, and testable transitions (Kazdin, 2007), a more precise account of attentional timing may clarify relapse clustering and substance-focused engagement beyond global distress or cue-based formulations.

### 1.2 Distinguishing SDM from adjacent accounts

The Strategic Distraction Motive (SDM) is proposed to address this timing gap. SDM does not dispute reinforcement-based, cue-reactivity, or affect-regulation accounts of relapse. Its contribution is narrower and process-specific: to formalize a within-person transition condition, perceived insolubility, under which attention reorganizes into addiction-related domains. The core claim is timing- and content-specific: addiction-related attentional pivots are hypothesized to occur when engagement with activated experience is appraised as regulatorily infeasible and to be detectable as measurable shifts in attentional and interactional organization rather than inferred motivational states. SDM therefore specifies the regulated medium as attentional organization rather than affect reduction. Whereas self-medication and experiential avoidance models predict increased substance use as a function of distress and efforts to reduce aversive affect (Aldao et al., 2010; Khantzian, 1997), SDM predicts that addiction-related attentional pivots occur when engagement is appraised as nonviable, even at moderate levels of affect, and that these pivots preferentially recruit addiction-specific attentional structure rather than generic distraction (Gross, 2015; Sheppes et al., 2011). This yields discriminant predictions beyond negative affect magnitude, craving intensity, cue exposure, and global experiential avoidance.

In contrast to incentive-sensitization and attentional-bias models, which describe cue-driven capture of attention through sensitized reward systems (Robinson and Berridge, 2008; Koob and Volkow, 2016), SDM predicts that attentional pivots may be triggered by meaning-level or relational appraisals in the absence of proximal drug cues and expressed in observable discursive and interactional shifts (e.g., topic narrowing, script shifts, changes in interpersonal agenda). Recent dynamic-systems and interpersonal-process work is broadly consistent with the view that short-term behavioral transitions are shaped by interacting within-person and relational processes rather than cue exposure alone (Gauld et al., 2025). Critically, SDM operates at the within-person level. Trait risk models identify between-person vulnerabilities such as insecure attachment or low perceived control (Mikulincer and Shaver, 2016). In contrast, SDM specifies a within-person state process that generates lagged predictions about imminent attentional reorganization, requiring disaggregation of within- and between-person effects in longitudinal models (Curran and Bauer, 2011; Tasca and Gallop, 2017).

Formally, SDM specifies a temporally ordered within-person sequence in which insolubility appraisals at time *t* predict attentional reallocation at time *t* + 1, which in turn predicts relapse risk or interactional restructuring at time *t* + 2, with attentional substitution operationalized as a within-person mediator in longitudinal models. The hypothesized sequence is expected to unfold across short intervals (e.g., minutes to hours in ecological momentary assessment; conversational turns in psychotherapy transcripts), requiring fine-grained temporal sampling to adjudicate directionality. Failure to observe such incremental, temporally ordered effects would argue against the added precision of the model.

## 2 Conceptual framework: insolubility and attentional governance

The Strategic Distraction Motive is grounded in the observation that distress does not uniformly recruit regulatory response. Individuals may tolerate intense affect when it is experienced as actionable, symbolizable, or relationally safe, while comparatively milder experiences may precipitate abrupt regulatory shifts when they are appraised as unmanageable. SDM formalizes this distinction through the construct of perceived insolubility, proposing that attention may function as a regulatory medium when engagement with activated experience is appraised as exceeding available symbolic, regulatory, or relational capacity.

SDM does not treat attentional substitution as a default response to distress. Rather, it specifies a transition condition (perceived insolubility) that can be operationalized and tested against adjacent explanations (e.g., affect magnitude, craving intensity, global experiential avoidance) using within-person process models (Curran and Bauer, 2011). This section defines perceived insolubility, distinguishes it from affect intensity, and situates attentional governance as a regulatory transition under appraisal-based constraint.

### 2.1 Perceived insolubility as a constraint on regulatory appraisal

Perceived insolubility is defined as a state-dependent appraisal that an activated experience cannot be effectively engaged, articulated, or resolved within current capacities. It does not denote objective impossibility or overwhelm; it reflects a momentary judgment that available regulatory moves (e.g., action, reappraisal, integration, disclosure) would be destabilizing. This distinguishes insolubility from distress intensity, stress exposure, or trauma activation per se. Emotion-regulation research demonstrates that regulatory strategy selection depends not solely on affect magnitude but also on perceived feasibility and cognitive cost (Gross, 2015; Sheppes et al., 2011). Accordingly, intense affect may remain manageable when coping options or relational containment are available, whereas less intense experiences may become insoluble when they threaten identity coherence, relational standing, or symbolic integration. For analytic clarity, perceived insolubility can be decomposed into three partially dissociable components:

- *Low perceived controllability*: no available action is experienced as capable of altering the core threat or its implications.
- *Low symbolizability*: difficulty sustaining reflective contact with the experience or integrating it into coherent narrative.
- *High anticipated relational cost*: expectation of shame, rejection, or loss of standing if the experience is engaged interpersonally.

Operationally, insolubility is the convergence of constraints sufficient to render engagement-oriented regulation momentarily nonviable. It is thus a constraint on regulation rather than a property of the stimulus. This distinguishes it from adjacent constructs: distress intolerance concerns the capacity to endure aversive affect; experiential avoidance concerns disengagement from unwanted internal experience; low perceived control indexes ineffective action; and relational threat concerns anticipated interpersonal consequences. Perceived insolubility refers to the point at which such constraints converge at the level of regulatory feasibility.

A boundary case clarifies the distinction. An individual may experience intense distress during an interpersonal conflict yet remain engaged if the situation feels controllable, articulable, or relationally safe. Conversely, more moderate distress may become insoluble when it threatens identity coherence or relational standing in ways that cannot be readily articulated or acted upon. In such cases, disengagement reflects not affective magnitude but the perceived nonviability of engagement-oriented regulation. This formulation also reframes classical notions of defense at the level of attentional organization: what is limited is not knowledge itself, but the range of meanings that can be actively maintained in awareness without destabilization (Vaillant, 1995; Cramer, 2006).

For perceived insolubility to function as a distinct construct, it must demonstrate incremental within-person predictive validity beyond adjacent processes, including negative affect, craving, perceived control, and experiential avoidance, in temporally ordered longitudinal models (Tasca and Gallop, 2017). It is best understood as a state-dependent configuration crossing a functional threshold, rather than a single latent factor or additive composite. Failure to show such convergence and incremental effects would indicate construct redundancy.

### 2.2 Attentional governance as a regulatory shift under constraint

When experiences are appraised as manageable, regulation typically proceeds via engagement-oriented strategies such as problem solving, cognitive reappraisal, meaning-making, or relational negotiation. These strategies presuppose adequate attentional bandwidth, symbolic capacity, and interpersonal safety. When engagement is appraised as nonviable, SDM proposes that regulation may shift toward attentional governance, defined as selective amplification and suppression of awareness within the broader architecture of emotion regulation (Gross, 2015).

In this frame, attention functions as a late-stage regulatory frontier: when action and meaning-making are judged nonviable, stability is preserved through selective governance of awareness. Attentional governance refers to measurable reallocations of cognitive and interactional priority (e.g., topic persistence, semantic narrowing, planning sequences), rather than subjective reports of distraction alone.

This extends process models showing that attentional deployment is preferentially selected when cognitive load is high or reappraisal is costly (Sheppes et al., 2011). SDM adds that, under perceived insolubility, attentional substitution may become recurrent and domain-specific. Attention is not merely redirected away from distress; it may be reallocated toward a bounded domain that constrains what is attended to, elaborated, and interpersonally negotiated. To preserve empirical tractability, SDM treats this shift as a measurable transition rather than an inferred motivational state, consistent with mechanism-focused standards (Nock, 2007).

### 2.3 Selection of addiction-related domain as attentional substitute

SDM does not assume that any distraction suffices under insolubility. Rather, it specifies a selection principle: addiction-related domains may be preferentially recruited when they provide advantages over alternative forms of attentional disengagement.

- First, addiction-related routines are highly structured. Substance use often unfolds through predictable sequences (anticipation, acquisition, consumption, recovery, recommitment) that narrow temporal focus and reduce uncertainty (Everitt and Robbins, 2016).
- Second, addiction is reinforced and learned. Repeated pairing of addiction-related routines with temporary stabilization increases the likelihood that substance-related cues and scripts will be recruited again, consistent with reinforcement-based accounts of addiction (Everitt and Robbins, 2016).
- Third, addiction is socially legible. Framing distress in terms of “the problem of addiction” converts diffuse suffering into a culturally recognizable narrative that can elicit predictable interpersonal responses (Goffman, 1959).
- Fourth, addiction affords constrained agency, providing an available course of action when no effective action is perceived with respect to the primary threat.

These features define a constrained selection process shaped by prior learning and current appraisal. Addiction-related attention should be recruited only when it provides greater structure, reinforcement value, accessibility, or social legibility than competing alternatives (e.g., rumination, work over-engagement, digital distraction). The attentional pivot is therefore not reflexive but context-sensitive.

### 2.4 From defense to attentional and intersubjective organization

SDM reframes classical notions of defense at the level of attentional organization. Psychodynamic traditions conceptualize denial and avoidance as protective responses to threat (Vaillant, 1995; Cramer, 2006). SDM aligns with this functional view but shifts unit of analysis from inferred content exclusion to constraint on attentional scope. Individuals may retain factual awareness while being unable to sustain attention to an experience’s implications without destabilization. What is regulated is not knowledge itself, but the range of meanings that can be actively maintained in awareness. This reframing permits *in vivo* study through observable markers such as topic-shift latency, discourse constriction, and sequential interaction patterns, rather than relying solely on retrospective inference.

A defining feature of SDM is its intersubjective scope. Attentional shifts toward addiction-related content may reorganize the relational field by shaping what others attend to, inquire about, and respond to. Attachment research indicates that relational activation can modulate regulatory strategy selection (Mikulincer and Shaver, 2016). Under conditions of increased closeness, evaluation, or anticipated loss, activated schemas may be appraised as insoluble. From this perspective, addiction-related discourse functions as an interactional organizer rather than merely a private distraction. It allows individuals to remain present in the relational field while modulating vulnerability and proximity through attentional narrowing rather than withdrawal. SDM therefore conceptualizes substance-related attentional substitution as an attention-mediated proximity-regulation strategy. Empirically, this yields testable predictions: addiction-framed pivots should reduce exploration of primary threat material and increase structured responses such as concern, advice, monitoring, or treatment talk.

### 2.5 Summary

The conceptual framework of SDM rests on four bounded claims:

1. Perceived insolubility increases the likelihood of transition from engagement-oriented regulation to attentional governance.
2. Attentional governance functions as a late-stage regulatory medium when engagement is appraised as nonviable.
3. Addiction-related domains may be preferentially recruited as substitute attentional structures due to their structure, reinforcement history, social legibility, and affordance of constrained agency, without assuming universality.
4. These shifts reorganize both intrapsychic processing and interpersonal interaction.

Together, these propositions define a process-level mechanism that is conceptually integrative, bounded in scope, clinically intelligible, and amenable to empirical test in temporally ordered within-person designs (Nock, 2007).

## 3 The strategic distraction motive: core mechanism and process model

Building on the preceding framework, this section specifies the Strategic Distraction Motive (SDM) as a process-level mechanism through which addiction-related behavior and discourse function as context-sensitive attentional regulation under perceived insolubility. SDM is defined, distinguished from adjacent constructs, and articulated as a recurrent regulatory cycle with identifiable stages, moderators, and boundary conditions consistent with mechanism-focused theory development (Kazdin, 2007).

### 3.1 Definition and functional architecture of SDM

The Strategic Distraction Motive refers to a state-dependent process in which attention is reallocated, under conditions of perceived insolubility, from engagement with destabilizing experience toward a structured and socially legible addiction-related domain. The pivot may involve substance use, addiction-related behavior, or addiction-focused discourse, but its defining feature is the reorganization of attentional priority rather than consumption itself.

“Strategic” denotes functional organization under constraint rather than deliberate planning. The pivot may be rapid, learned, and only partially accessible to reflective report. SDM therefore does not frame addictive behavior as either pure habit or unconstrained choice. Instead, attentional substitution is conceptualized as a learned, context-sensitive regulatory strategy that can operate below full reflective awareness while remaining responsive to identifiable situational triggers such as proximity, shame exposure, identity threat, or relational evaluation. This placement is consistent with evidence that attentional strategies can modulate emotional processing without full deliberative awareness (Gross, 2015) and with defensive models that conceptualize avoidance as protective under conditions of limited regulatory capacity (Vaillant, 1995).

SDM conceptualizes attentional organization as the primary regulatory medium through which addiction-related engagement preserves short-term coherence under perceived insolubility (Khantzian, 1997; Koob and Volkow, 2016). SDM operates across two linked levels. Intrapsychically, attentional narrowing reduces the range of meanings that must be maintained simultaneously, preserving short-term coherence. Interpersonally, the addiction frame organizes the relational field by recruiting culturally recognizable scripts such as concern, monitoring, caretaking, or treatment planning (Goffman, 1959; Eubanks et al., 2018). These levels are mutually reinforcing: the same shift that reduces internal load also structures external responses. In this sense, SDM situates addiction-related behavior within an intermediate regulatory tier, functioning as a constrained adaptive response that preserves short-term coherence while deferring engagement with the primary threat.

### 3.2 Temporal dynamics: the SDM cycle

SDM specifies a recurrent regulatory sequence:

1. Activation of potentially insoluble material: relational, affective, or identity-linked material becomes salient, particularly under conditions of increased proximity, evaluation, or identity threat (Mikulincer and Shaver, 2016).
2. Appraisal of insolubility (*t*): the activated material is appraised, often rapidly and pre-reflectively, as unmanageable within current capacities, such that engagement-oriented regulation is experienced as ineffective or destabilizing (Gross, 2015).
3. Attentional reallocation (*t* + 1): attention shifts toward a structured addiction-related domain.
4. Temporary intrapsychic and interpersonal organization (*t* + 2): narrowed attention stabilizes experience and interaction.
5. Reinforcement: short-term organization strengthens the learned coupling between insolubility appraisal and attentional substitution.
6. Recurrence: similar pivots become more likely under comparable future conditions.

The critical transition is from appraisal to attentional reallocation. This transition reflects a learned, context-sensitive reorganization of attentional control rather than a fully deliberative shift, consistent with evidence that attentional deployment can operate efficiently with limited reflective access (Gross, 2015; Sheppes et al., 2011). This pivot is instantiated as a reconfiguration of attentional control: narrowing of attentional scope, reduced elaboration of the primary threat, and reallocation of cognitive and interactional priority toward a bounded substitute domain. Because SDM is formulated as a state-dependent within-person process rather than a fixed trait, its expression should vary across contexts as regulatory feasibility changes (Curran and Bauer, 2011).

**Figure 1:**
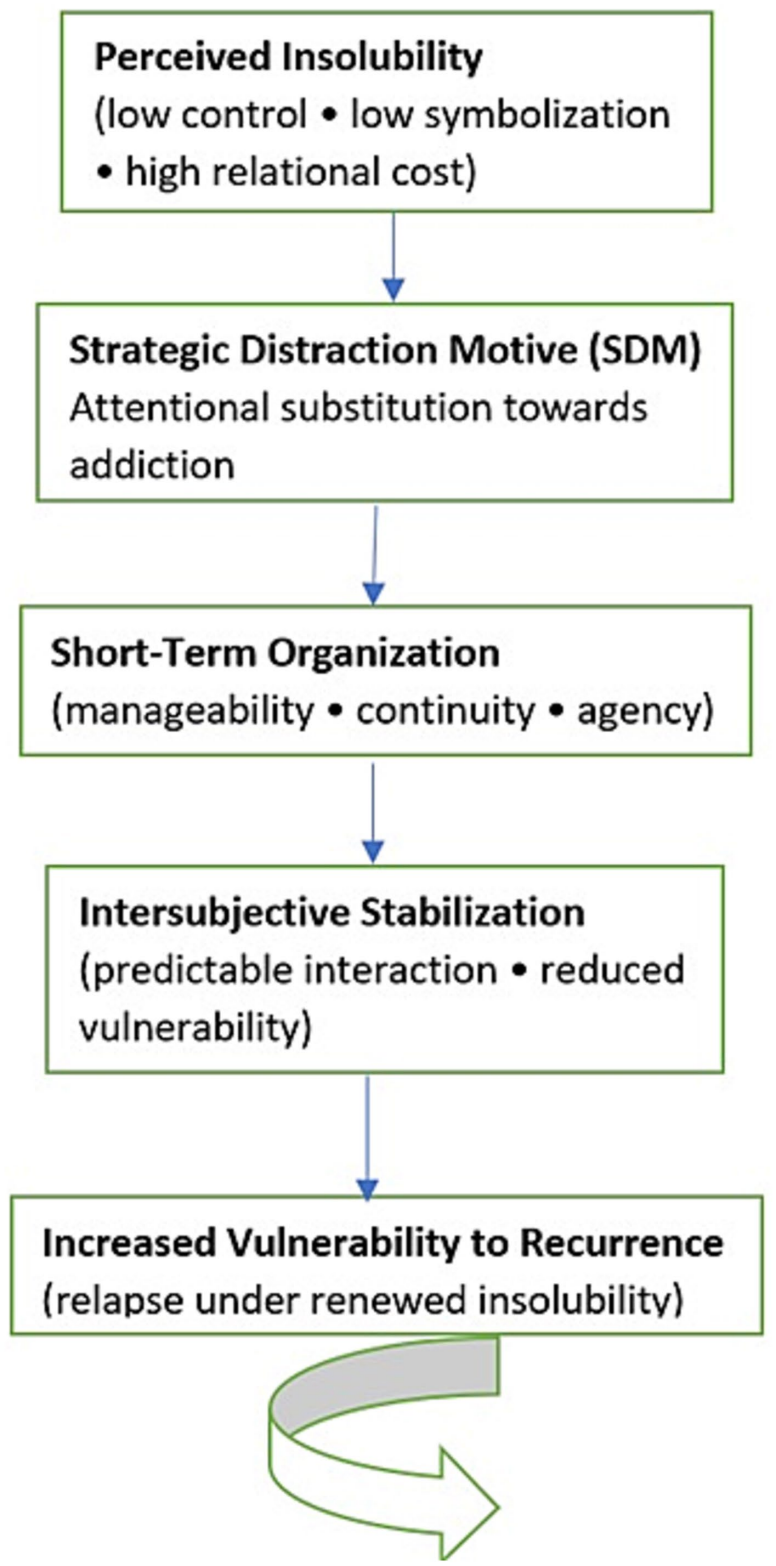
The strategic distraction motive (SDM): a recurrent attentional-regulation cycle. When experience is appraised as emotionally or existentially insoluble, attention is redirected away from destabilizing material toward a structured and socially legible addiction-related domain. This attentional substitution produces short-term experiential and interpersonal organization, which reinforces the coupling between insolubility appraisals and future attentional redeployment. The looped structure emphasizes recurrence rather than linear causation and situates relapse as reactivation of attentional substitution under renewed insolubility.

This mechanism helps explain why relapse may cluster around relational or meaning-level activation rather than stress magnitude alone, and why behavioral suppression without expanded regulatory capacity may be unstable (Marlatt and Donovan, 2005). Intensive longitudinal and dynamic relapse models similarly conceptualize lapse risk as time-varying within persons rather than as a static vulnerability. Reinforcement processes further strengthen the appraisal–substitution coupling over time, consistent with habit-learning and compulsivity models (Koob and Volkow, 2016). Unlike established relapse formulations that emphasize affect escalation, coping failure, and abstinence violation effects, SDM specifies attentional substitution as the mediating mechanism linking appraisal-based constraint to relapse clustering (Figure 1).

### 3.3 Moderators, boundary conditions, and falsifiability

The expression of SDM is moderated by factors that influence perceived insolubility and available containment, including attachment security, relational safety, alliance quality, coping resources, and alternative self-regulation repertoires (Mikulincer and Shaver, 2016; Horvath et al., 2011). As symbolic articulation, co-regulation, or tolerance for ambiguity increase, the appraisal of insolubility should weaken, reducing reliance on attentional substitution.

SDM is not intended to explain substance use driven primarily by pharmacological dependence, stable habit responding without attentional reorganization, or contexts in which precipitating experiences are appraised as manageable (Koob and Volkow, 2016). Nor does it claim that addiction-related attentional redirection is present in all individuals or episodes.

The model would be undermined if attentional shifts showed no temporal coupling to insolubility cues, failed to reorganize interpersonal responding, or did not improve prediction of relapse timing or interactional restructuring once established predictors such as craving, affect, and cue exposure were modeled (Witkiewitz and Marlatt, 2004). These falsification criteria align SDM with mechanism-focused theory development, which requires temporally ordered processes, observable mediating transitions, and incremental predictive validity rather than *post hoc* interpretation (Kazdin, 2007).

## 4 Positioning SDM relative to adjacent models: discriminant validity and scope

The Strategic Distraction Motive (SDM) is not proposed as an alternative etiology of addiction. It is a process-level specification of when and how attention reorganizes into addiction-related domains under constrained regulatory conditions. Its contribution therefore depends on two criteria: (a) non-redundancy with adjacent constructs, and (b) discriminant, falsifiable predictions testable using intensive within-person designs (Curran and Bauer, 2011; Kazdin, 2007). The sections below clarify SDM’s points of overlap and divergence relative to dominant models in addiction science and clinical psychology.

SDM is distinguished along three dimensions.

First, it specifies a timing-sensitive transition condition: attentional reallocation is predicted to occur when activated experience is appraised as regulatorily insoluble, rather than as a function of affect intensity, craving, or cue exposure alone.

Second, it identifies the regulated medium as attentional organization, rather than affect reduction, reward seeking, or impulse control.

Third, it predicts content-specific reallocation, such that attention preferentially shifts into addiction-related domains rather than generic distraction.

These commitments yield testable discriminant predictions: within individuals, perceived insolubility at time *t* should prospectively predict addiction-specific attentional pivots at time *t* + 1, which in turn predict relapse risk or interpersonal reorganization at time *t* + 2, above and beyond negative affect, craving, cue exposure, and experiential avoidance.

### 4.1 Relation to self-medication and negative reinforcement accounts

Self-medication models conceptualize substance use as affect reduction (Khantzian, 1997). SDM is compatible with this function but specifies a different regulated medium: attentional organization rather than affect magnitude. Under SDM, addiction-focused pivots are predicted when engagement is appraised as nonviable, even at moderate affect levels. Two experiences matched for negative affect should differ in pivot probability as a function of insolubility appraisal. If affect magnitude fully accounts for within-person pivot timing, SDM offers no incremental value.

### 4.2 Relation to experiential avoidance frameworks

Experiential avoidance frameworks conceptualize substance use as escape from unwanted internal experience (Hayes et al., 1996). Within this literature, avoidance is typically conceptualized as a broad functional process rather than as inherently content-specific. SDM overlaps functionally with avoidance accounts but introduces additional constraints regarding content specificity and micro-temporal transition conditions.

First, SDM predicts content specificity: attentional redirection preferentially recruits an addiction-related domain (e.g., use planning, recovery scripts, relapse self-talk) rather than neutral distraction. Avoidance accounts typically predict disengagement from aversive material but do not specify systematic reorganization into a culturally structured addiction frame.

Second, SDM predicts timing specificity: pivots occur as micro-temporal transitions following state appraisals of insolubility, rather than as diffuse correlates of generalized distress.

Third, SDM predicts interactional consequences: addiction-framed pivots reorganize interpersonal responding (e.g., shifts toward problem-solving or treatment scripts), extending beyond intrapsychic disengagement.

SDM would be supported if perceived insolubility prospectively predicts addiction-content pivots more strongly than non-addiction distraction within individuals. If redirection occurs equivalently across generic distraction domains under generalized distress, SDM would collapse into broader avoidance formulations.

### 4.3 Relation to craving, incentive-salience, and habit models

Neurobehavioral models emphasize cue-reactivity, incentive salience, habit learning, and impaired top–down control as central mechanisms of compulsive use (Everitt and Robbins, 2016; Robinson and Berridge, 2008). SDM does not dispute these mechanisms. Rather, it targets a complementary question: the within-person transition conditions under which attention reorganizes into addiction-related domains in relational or meaning-laden moments.

Attentional pivot in SDM is not defined as transient cue-driven attentional capture or craving elaboration. It refers to a structured reorganization of cognitive and discursive activity around addiction-related scripts that narrows alternative meaning elaboration and shifts interactional agendas. The defining feature is organizational: the addiction domain becomes the primary interpretive frame structuring subsequent thought or interaction.

SDM yields three discriminant predictions:

1. *Pivots without proximal drug cues*: addiction-focused shifts may occur following meaning-level or relational appraisals even when cue exposure is low.
2. *Discursive/interactional signature*: pivots manifest as observable reorganization of topic structure and interpersonal scripts, not merely attentional bias toward salient stimuli.
3. *Incremental timing prediction*: insolubility-linked pivots should improve prediction of near-term lapse timing beyond craving intensity and cue exposure in within-person models.

### 4.4 Relation to classical defense and denial

Psychodynamic traditions conceptualize denial and avoidance as protective responses to threat (Vaillant, 1995; Cramer, 2006). SDM aligns with this functional perspective but shifts the unit of analysis from inferred content exclusion to observable constraint on attentional scope. Under SDM, individuals may retain factual awareness while being unable to sustain attention to implications that threaten relational or identity stability. The emphasis therefore moves from retrospective inference of unconscious exclusion to measurable process markers (e.g., latency to topic shift, semantic narrowing), consistent with mechanism-focused standards emphasizing temporal ordering and observable transitions (Kazdin, 2007).

### 4.5 Relation to attachment-based models

Attachment research links insecurity to substance use vulnerability and demonstrates that relational activation shapes regulatory strategy selection (Mikulincer and Shaver, 2016). SDM refines this literature by specifying a within-person contingency: addiction-related attentional narrowing is expected to intensify during attachment activation only when the activated experience is appraised as insoluble. This conditional formulation yields a discriminant test. Contexts that increase perceived relational safety (e.g., secure-base cues, strong alliance conditions) should weaken the coupling between attachment activation and addiction-focused pivots, even when trait attachment insecurity remains stable.

### 4.6 Relation to identity and stigma models

Identity and stigma frameworks conceptualize addiction as a socially shaped role or self-concept with consequences for behavior and help-seeking (Goffman, 1959). SDM is compatible with this perspective but shifts the analytic emphasis from stable identity endorsement to situational deployment. Within SDM, addiction-related discourse functions as an attention-organizing frame selectively mobilized under perceived insolubility. The critical distinction is structural rather than symbolic: the addiction frame reorganizes interaction by narrowing topic exploration and activating culturally available scripts (e.g., problem-solving, treatment planning), thereby constraining engagement with destabilizing material.

The discriminant hinge is therefore interactional. If addiction-framed pivots reliably alter audience response patterns (e.g., reduce exploration of primary threat material while increasing structured intervention talk) relative to non-addiction framing of equivalent affective content, this would support SDM’s intersubjective mechanism.

### 4.7 Scope conditions and discriminant falsifiability

SDM is intentionally constrained. It is not intended to explain:

- substance use driven primarily by pharmacological dependence absent meaning-level threat,
- stable habitual responding without observable attentional or interactional reorganization,
- contexts in which precipitating experiences are appraised as workable or soluble.

A decisive test of SDM is not whether perceived insolubility correlates with addiction-related attention, but whether it explains within-person variance in the timing of addiction-focused pivots that remains after craving intensity, negative affect, cue exposure, and state experiential avoidance are modeled. Using intensive longitudinal data and person-mean centering to disaggregate within- and between-person effects (Tasca and Gallop, 2017), SDM predicts that insolubility at time *t* will prospectively predict addiction-content pivots at time *t* + 1 above established predictors. Cross-lagged and dynamic structural equation models should additionally evaluate bidirectional effects to determine whether insolubility prospectively predicts addiction-focused pivots beyond reverse pathways.

The model’s incremental contribution would be undermined if:

(a) perceived insolubility does not prospectively predict addiction-content pivots within individuals,
(b) pivots show no content specificity relative to generic distraction, or
(c) pivots fail to improve prediction of near-term lapse timing once craving, negative affect, and cue exposure are modeled.

Failure to observe such incremental, temporally ordered, and content-specific effects would argue for redundancy with existing models.

### 4.8 Summary: core novelty

SDM does not propose a new motive for substance use, nor does it displace reinforcement, cue-reactivity, or negative affect accounts. Its contribution is to formalize a within-person transition condition (perceived insolubility) under which attention reorganizes into addiction-related domains. It specifies the regulated medium as attentional organization rather than affect reduction, predicts observable pivots in topic focus and interactional structure rather than inferred motivational states, and yields timing-sensitive, content-specific, and falsifiable predictions. Its novelty lies in specifying a process-level mechanism with discriminant commitments, not in relabeling avoidance (Cronbach and Meehl, 1955; Kazdin, 2007; Table 1).

**Table 1:** Summary: core novelty.

Model	Core regulatory logic	What triggers the shift?	SDM’s distinctive claim	Falsifiable prediction
Self-medication/negative reinforcement	Substance use reduces painful affect or psychological distress.	Negative affect, distress, emotional strain.	SDM treats attentional organization, rather than affect reduction, as the primary regulated process. Addiction-focused pivots occur when engagement is appraised as insoluble, even at moderate affect levels.	Perceived insolubility should predict addiction-focused pivots above negative affect intensity alone.
Experiential avoidance	Substance use functions as disengagement from unwanted internal experience.	Aversive thoughts, emotions, bodily states.	SDM predicts content-specific attentional reallocation into addiction-related domains rather than generalized distraction or disengagement.	Insolubility should preferentially predict addiction-related pivots over neutral distraction within persons.
Cue-reactivity/incentive-salience/habit models	Drug-related cues activate sensitized reward-learning and habit systems that bias attention toward craving and substance-seeking behavior.	Cue exposure, sensitized reward salience, conditioned reinforcement, habit activation.	SDM predicts that attentional reallocation can be initiated by appraisal of relational or symbolic insolubility even in the absence of proximal drug cues or elevated cue salience.	Addiction-focused pivots should occur following relational or meaning-level insolubility even under low-cue conditions and should explain variance beyond cue exposure and craving intensity.
Defense/denial models	Defensive processes protect against destabilizing awareness or affect.	Shame, conflict, threat to self or attachment.	SDM operationalizes defense as observable attentional narrowing and interactional reorganization rather than inferred unconscious exclusion.	Pivots should be detectable through measurable markers such as topic shifts, semantic narrowing, and altered interactional sequencing.
Identity/stigma models	Addiction-related identity frames organize self-appraisal and interpersonal behavior through socially reinforced role expectations and stigma processes.	Identity threat, shame exposure, social evaluation, anticipated stigma.	SDM conceptualizes addiction discourse as a situationally deployed attentional-organizing frame that constrains cognitive and interactional processing under perceived insolubility rather than as a stable identity structure.	Addiction-framed pivots should systematically redirect interaction toward structured addiction scripts (e.g., monitoring, treatment planning, risk management) while suppressing elaboration of primary threat material beyond effects of distress or stigma alone.
Attachment-based models	Regulatory responses organize proximity, safety, and attachment security.	Attachment activation, insecurity, relational threat.	SDM specifies a conditional within-person process: attachment activation alone is insufficient; pivots are predicted specifically when activation is appraised as regulatorily insoluble.	Increased relational safety should weaken the coupling between attachment activation and addiction-focused pivots.
Strategic distraction motive (SDM)	Addiction-related behavior/discourse functions as attentional substitution under perceived insolubility.	Appraisal that engagement is regulatorily nonviable, unsymbolizable, or relationally unsafe.	SDM specifies a timing-sensitive, content-specific, within-person mechanism linking insolubility appraisal → attentional pivot → relapse/interpersonal reorganization.	Insolubility at time *t* should prospectively predict addiction-specific attentional pivots at time *t* + 1, beyond affect, craving, cue exposure, and experiential avoidance.

## 5 Clinical implications: translating strategic distraction without reification

Because this article advances a process-level mechanism rather than a treatment package, the clinical implications of the Strategic Distraction Motive (SDM) concern mechanism translation rather than prescriptive technique. SDM does not introduce a new therapy model; rather, it reframes how clinicians may conceptualize substance use, relapse, and apparent resistance when addiction-related attentional substitution is operative. The implications follow directly from SDM’s central claim: when activated experience is appraised as regulatorily insoluble, attentional organization may shift toward addiction-related domains to preserve short-term experiential and interpersonal coherence. This formulation is consistent with evidence that attentional deployment functions as a regulatory strategy within emotion-regulation systems (Gross, 2015) and with neurocognitive models indicating that addiction involves altered interactions between salience attribution and executive control networks governing attention and behavioral prioritization (Goldstein and Volkow, 2011).

### 5.1 From symptom suppression to attentional governance

Within SDM, substance use is conceptualized as a regulatory solution under constraint rather than solely as a target symptom. When addiction-related behavior or discourse preserves organization under perceived insolubility, interventions focused narrowly on behavioral suppression may destabilize the regulatory structure the behavior temporarily maintains. Process research links shame escalation, alliance strain, and rupture mismanagement to disengagement and poorer outcomes (Eubanks et al., 2018). Relapse research similarly indicates that punitive responses to lapses predict worse trajectories than learning-oriented responses (Marlatt and Donovan, 2005).

From an SDM perspective, the task shifts from eliminating distraction to expanding regulatory feasibility. The operative question becomes: What experience is currently appraised as unmanageable without attentional substitution? This reframing maintains accountability while situating behavioral change within expanded symbolic, relational, and regulatory capacity.

### 5.2 Functional validation and alliance stabilization

If attentional substitution manages perceived insolubility, premature confrontation (e.g., labeling behavior as “avoidance”) may intensify threat. Alliance rupture research shows that misattuned interventions amplify defensive responses and disengagement (Eubanks et al., 2018). Within SDM, validation stabilizes the relational field without endorsing substance use. Acknowledging the regulatory function of the behavior may reduce automatic pivoting and render attentional shifts observable rather than reflexively enacted. Attachment-informed findings indicate that perceived relational safety facilitates regulatory flexibility and exploratory engagement (Mikulincer and Shaver, 2016).

Only when substitution is no longer required for immediate stability does reflective examination become tolerable. Validation thus functions as a precondition for modifying the mechanism.

### 5.3 Targeting insolubility rather than use

SDM shifts therapeutic focus from substance use to the appraisal conditions precipitating substitution. Clinically, this involves identifying which dimension of perceived insolubility is active:

- Low perceived controllability
- Low symbolizability
- High anticipated relational cost

Interventions that increase perceived solvability-through scaffolding articulation, strengthening relational safety, or graded engagement-directly target the transition condition. Regulatory flexibility research suggests that adaptive functioning depends more on feasibility calibration than global strategy suppression (Aldao et al., 2010).

SDM thus predicts that relapse vulnerability may persist while insolubility appraisals remain intact, regardless of insight or declared motivation. Symptom reduction without expanded feasibility is therefore unlikely to endure.

### 5.4 Co-regulation and graduated engagement

Because attentional substitution preserves short-term organization, intervention may require graded engagement with threatening material rather than direct confrontation. Emotion-regulation research indicates that strategy selection depends on perceived feasibility and cognitive load (Sheppes et al., 2011). Clinically, pacing should increase capacity for symbolization and relational exposure while maintaining sufficient containment. As perceived feasibility increases, insolubility appraisals should weaken, reducing reliance on addiction-focused pivots. Progress is indexed by greater flexibility in attentional allocation and reduced automaticity of substitution.

### 5.5 Reframing relapse as process signal

SDM conceptualizes relapse as reactivation of attentional substitution under renewed insolubility rather than simple motivational failure. This aligns with dynamic relapse prevention models (Witkiewitz et al., 2013). Clinically, relapse should prompt inquiry into what became insoluble at that moment and how substitution restored short-term organization. This framing preserves responsibility while reducing shame-based disengagement.

### 5.6 Ethical positioning: function without endorsement

Acknowledging regulatory function must not be confused with justifying substance use. SDM distinguishes the function of the behavior, preserving coherence under insolubility, from endorsement of its form. This distinction permits accountability without moralization. Within SDM, compassion is mechanism-informed: understanding the process enables targeted modification of the transition condition without collusion with harmful behavior.

### 5.7 Summary

The clinical implications derive directly from SDM’s mechanism:

1. Substance use may function as attentional organization under perceived insolubility.
2. Interventions that ignore this function risk destabilizing short-term coherence.
3. Expanding perceived solvability reduces reliance on substitution.
4. Relapse signals reactivation of the transition condition.
5. Compassion enhances alliance stability without excusing harm.

These implications do not constitute a new treatment model. They translate a timing-sensitive, content-specific attentional mechanism into process-informed clinical reasoning aimed at increasing precision in case formulation and intervention timing.

## 6 Empirical tractability, operationalisation, and falsifiability

The Strategic Distraction Motive is formulated as a process-level mechanism whose empirical value depends on whether it yields incremental, temporally ordered, and content-specific prediction beyond established constructs. Its central empirical claim is not that perceived insolubility correlates with distress or substance use, but that it predicts a specific within-person transition: from engagement with activated experience to addiction-focused attentional reallocation, followed by behavioral or interpersonal consequences. In this sense, SDM represents a theory-building proposal that derives empirically testable predictions from a clinically specific process model (for a general overview, see Gelo et al., 2020a, 2020b).

### 6.1 Core discriminant hypotheses

SDM generates three discriminant hypotheses.

First, perceived insolubility should prospectively predict addiction-focused attentional pivots within individuals. Momentary increases in perceived insolubility should predict subsequent shifts toward addiction-related cognition, behavior, or discourse above and beyond negative affect, craving intensity, cue exposure, perceived control, and experiential avoidance. These pivots should in turn predict near-term lapse risk, urge escalation, or relapse clustering.

Second, attentional pivots should show content specificity. Under comparable levels of affective arousal or stress, perceived insolubility should preferentially predict addiction-related shifts, such as use planning, craving elaboration, relapse self-talk, or addiction-focused discourse, rather than generic distraction or unrelated disengagement. If attentional redirection distributes equivalently across domains, SDM would collapse into a broader avoidance or distraction account.

Third, addiction-framed pivots should reorganize interpersonal responding. When attention shifts into addiction-related content, interaction should become structured around culturally legible addiction scripts, including concern, advice, monitoring, treatment discussion, or problem-solving, while reducing exploration of the primary threat material. These effects should not be fully attributable to affect intensity or general distress.

### 6.2 Operationalising perceived insolubility

Perceived insolubility should be assessed as a state-dependent appraisal rather than a trait vulnerability. In ecological momentary assessment, psychotherapy process research, or experimental paradigms, it can be indexed through brief measures of three partially dissociable components:

1. *Perceived controllability*: the extent to which the person experiences any available action as capable of influencing or resolving the situation.
2. *Symbolizability*: the extent to which the person can think about, articulate, or narratively organize the activated experience.
3. *Anticipated relational cost*: the extent to which engaging or disclosing the experience is expected to produce shame, rejection, loss of standing, or relational destabilization.

These components may be modeled separately and as a convergent state configuration. SDM would be supported if their convergence improves prediction of attentional reallocation beyond negative affect, craving, perceived control alone, and experiential avoidance. The construct would be weakened if its predictive effects are fully absorbed by these adjacent variables or if the proposed convergence adds no incremental value.

### 6.3 Operationalising attentional reallocation

Attentional reallocation refers to a measurable shift in cognitive, behavioral, or interactional priority from the primary activated experience toward an alternative domain. In SDM, the key outcome is not distraction per se, but addiction-specific attentional substitution. Process research based on the analysis of psychotherapy session transcript provides an established basis for modeling such shifts as sequential linguistic and interactional patterns over time (e.g., de Felice et al., 2019, 2020).

This can be assessed through:

- EMA items capturing momentary shifts in attentional focus, including substance-related planning, craving elaboration, recovery discourse, or relapse-related rumination;
- behavioral and linguistic indicators such as topic-shift latency, semantic narrowing, repeated return to addiction-related themes, or reduced elaboration of primary threat material;
- optional convergent measures such as task-switching latency, eye-tracking, passive sensing, or digital trace indicators where appropriate.

Crucially, attentional reallocation should be modeled as a within-person transition rather than a static state. The relevant question is whether perceived insolubility at time *t* predicts addiction-specific attentional reallocation at time *t* + 1.

### 6.4 Testing content specificity

Content specificity is central to SDM’s discriminant validity. Empirical tests should compare addiction-related pivots with non-addiction distraction domains, such as neutral activities, digital distraction, work over-engagement, generalized rumination, or unrelated topic shifts.

The model predicts that, under matched affective and contextual conditions, perceived insolubility will more strongly predict addiction-related reallocation than generic distraction. This claim is falsifiable: if insolubility predicts attentional disengagement broadly without preferential recruitment of addiction-related domains, SDM would be better understood as a variant of generalized avoidance.

### 6.5 Testing intersubjective reorganization

SDM predicts that attentional substitution reorganizes interpersonal responding. This can be examined through self-report, observational coding, psychotherapy transcripts, dyadic interaction paradigms, or experimental vignette designs.

Relevant indicators include:

- reduced elaboration of the primary relational, symbolic, or identity-linked threat;
- increased addiction-focused problem solving, monitoring, reassurance, or treatment planning;
- shifts in conversational contingencies or interactional agenda following addiction-framed pivots.

A strong test would compare responses to addiction-framed versus non-addiction-framed disclosures matched for affective intensity. SDM would be supported if addiction framing systematically redirects interpersonal attention toward addiction-related scripts beyond what is explained by distress alone. The intersubjective component would be weakened if these shifts are absent or fully attributable to affect intensity.

### 6.6 Integrated within-person test

The strongest empirical test of SDM is a temporally ordered within-person model in which:

- perceived insolubility at time *t*
- predicts addiction-specific attentional reallocation at time *t* + 1; which in turn
- predicts subsequent behavioral or interpersonal outcomes at time *t* + 2.

Behavioral outcomes may include urge escalation, substance use, lapse risk, or relapse clustering. Interpersonal outcomes may include increased monitoring, structured concern, or reduced exploration of primary threat material.

Analyses should disaggregate within- and between-person variance and test incremental prediction beyond negative affect, craving, cue exposure, perceived control, and experiential avoidance. Cross-lagged, multilevel, dynamic structural equation, or intensive longitudinal mediation models should additionally evaluate reverse pathways, including whether craving or prior addiction-focused attention predicts later insolubility appraisals.

Support for SDM requires evidence that:

1. perceived insolubility prospectively predicts attentional reallocation;
2. reallocation is preferentially addiction-related rather than generically distracting;
3. the sequence improves prediction beyond established affective, cue-based, and avoidance mechanisms;
4. attentional reallocation predicts downstream behavioral or interpersonal outcomes.

Failure to observe these temporally ordered, content-specific, and incremental effects would argue against the model.

### 6.7 Scope conditions and transdiagnostic boundary

SDM is not intended to explain all substance use. Its scope is limited to episodes characterized by appraisal-based constraint and attentional substitution. It is not designed to account for substance use driven primarily by pharmacological dependence, stable habit responding without observable attentional reorganization, or contexts in which precipitating experiences are experienced as workable or soluble.

The model may have transdiagnostic implications. Similar patterns of attentional substitution may occur in eating disorders, compulsive caregiving, work over-engagement, or other forms of maladaptive regulation where attention narrows toward a structured domain under identity or relational threat. However, the present account is intentionally restricted to addiction. Substance use represents a particularly potent form of attentional substitution because pharmacological reinforcement, routinized behavioral sequences, and social legibility make addiction-related domains unusually effective organizers of attention and interaction. This boundary preserves specificity and prevents SDM from collapsing into a general theory of avoidance.

## 7 Conclusion

The Strategic Distraction Motive offers a process-level account of addiction-related engagement as attentional regulation under perceived insolubility. Its central claim is deliberately narrow: rather than proposing a new global theory, SDM specifies a within-person transition condition that may help explain why addiction-related pivots cluster around particular relational, symbolic, or identity-threatening moments rather than around distress magnitude alone.

The framework’s value depends on empirical discriminability. SDM should be supported only if perceived insolubility prospectively predicts addiction-specific attentional reallocation beyond negative affect, craving, cue exposure, perceived control, and experiential avoidance; if these shifts show content specificity relative to generic distraction; and if they produce measurable interpersonal reorganization. Conversely, the model would be weakened if attentional shifts occur independently of insolubility, distribute equivalently across distraction domains, or fail to improve prediction of relapse timing and interactional change beyond established predictors.

Clinically, SDM reframes addiction-related behavior as a constrained regulatory solution rather than solely a symptom to suppress. This does not justify harmful behavior, but highlights why interventions focused only on behavioral control may be unstable when insolubility appraisals persist. The implication is not a new treatment model, but greater precision in case formulation: expanding perceived solvability, symbolic articulation, and relational safety so that engagement-oriented regulation becomes viable.

## Data Availability

The original contributions presented in the study are included in the article/supplementary material, further inquiries can be directed to the corresponding author.
